# Noma staging: a review

**DOI:** 10.1186/s41182-022-00431-6

**Published:** 2022-06-13

**Authors:** Razia Abdool Gafaar Khammissa, Johan Lemmer, Liviu Feller

**Affiliations:** 1 Department of Periodontics and Oral Medicine, School of Dentistry, Faculty of Health Sciences, University of Pretoria, Pretoria, South Africa; 2 Department of Oral Medicine and Periodontology, University of the Witwatersrand, Johannesburg, South Africa; 3 111 Portman Place, Fir Avenue, Bantry Bay, Cape Town, 8005 South Africa

**Keywords:** Noma classification, Acute noma, Arrested noma, Necrotising gingivitis, Necrotising periodontitis, Necrotising stomatitis

## Abstract

Noma is a bacterial, non-communicable, grossly destructive and disfiguring necrotising oro-facial disease. It is rare, but occurs most commonly in chronically malnourished children with other debilitating illnesses, in remote, poverty-stricken communities, mainly in sub-Saharan Africa, and much more rarely in central Latin America and in parts of Asia. In South Africa and in Zimbabwe, noma is observed, again rarely, in immunosuppressed HIV-seropositive subjects. The World Health Organization (WHO) has classified noma into five sequential stages: stage 1, acute necrotising ulcerative gingivitis; stage 2, oedema; stage 3, gangrene; stage 4, scarring; stage 5, sequela. In the opinion of the authors, this WHO classification requires fundamental re-appraisal. The purpose of this viewpoint article is to highlight the weaknesses of this classification, and to propose a simpler, more logical and practical evidence-based staging of noma, which if used should improve the quality and value of future epidemiological data about noma.

## Introduction

Noma is a non-communicable, non-recurrent oro-facial necrotising infection, affecting mainly debilitated, malnourished children in remote rural, poverty-stricken communities in sub-Saharan Africa [1–8] and in Asia [7, 9, 10]. It may affect also HIV-seropositive immunosuppressed subjects [5, 11, 12] and rarely HIV-seronegative, impoverished, malnourished subjects of any age, anywhere in the world [12–14]. Noma is sometimes misleadingly and erroneously referred to ‘cancrum oris’. This is incorrect because the term cancrum oris that originates from Latin, meaning oral cancer, while in fact noma is a non-cancerous, necrotising oro-facial disease [15, 16].

Noma runs a fulminating clinical course causing severe facial destruction, and without prompt treatment the disease is fatal in up to 90% of cases [14, 17]. If the acute destructive phase of noma halts, either spontaneously or in response to treatment, there is a phase of healing and repair; but most survivors suffer gross facial disfigurement, severe functional impairment and gravely adverse psychological and social consequences [5, 8, 17, 18] (Fig. 5).

It is believed that noma is an opportunistic infection caused mainly by the commensal anaerobic microorganisms within polybacterial dento-gingival plaques. It occurs in most cases in children living in unhygienic, disease-promoting environments. Severe malnutrition and immune suppression appear to be the predominant systemic risk factors [19, 20].

According to [1, 21, 22] and the World Health Organization [7, 23] noma can be classified into five stages:

- Stage 1: acute necrotising ulcerative gingivitis.
- Stage 2: oedema.
- Stage 3: gangrene.
- Stage 4: scarring.
- Stage 5: sequela.

It is the opinion of the authors that this classification requires fundamental revision since in its current form it impedes efforts to understand the aetiopathogenesis of, and successfully prevent and treat this devastating neglected disease.

### Noma precursors

**Fig. 1:**
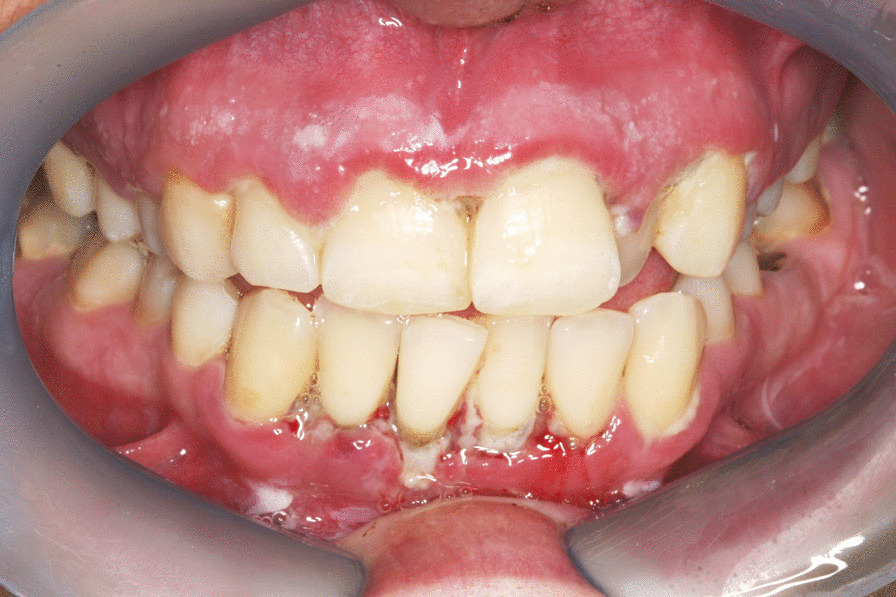
Necrotising gingivitis adult HIV-seropositive subject: gingival marginal necrosis and truncation of interdental papillae

**Fig. 2:**
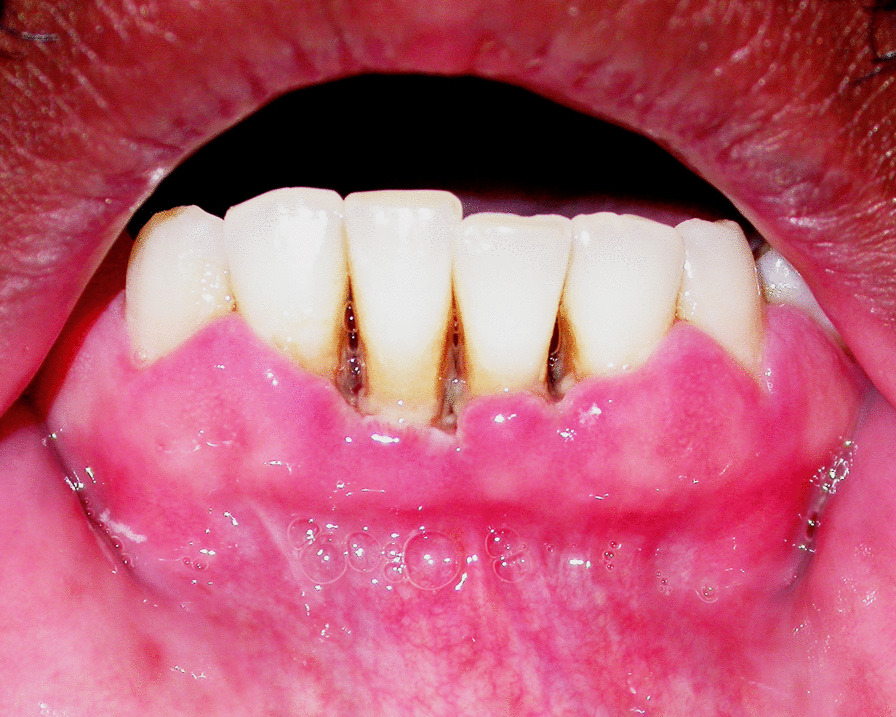
Necrotising periodontitis adult seropositive subject: necrotic gingiva has separated; necrosis advancing into periodontium

**Fig. 3:**
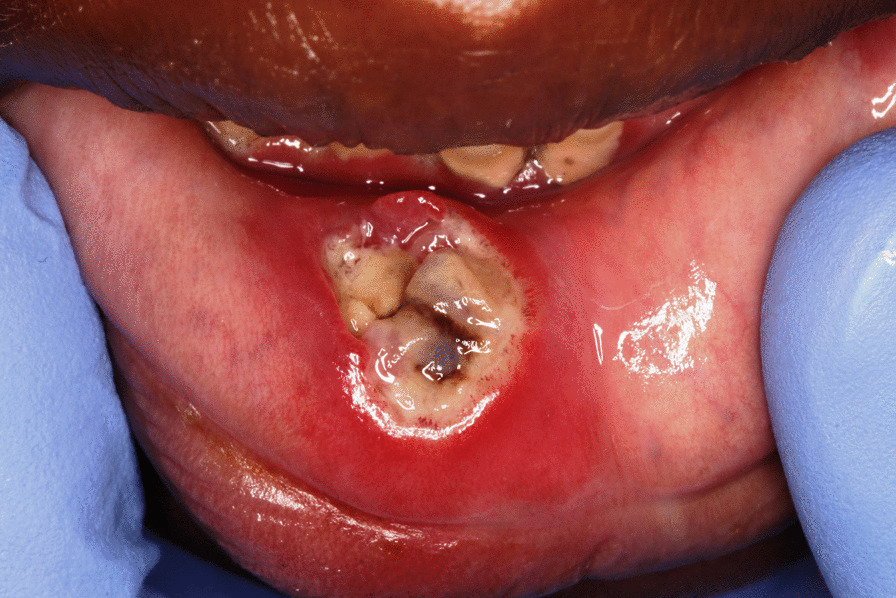
Necrotising stomatitis lower labial mucosa, following necrotising gingivitis: ‘contact’ spread: young HIV-seropositive male

Noma starts in the mouth as bacteria-induced necrotising gingivitis that progresses to necrotising periodontitis and then to necrotising stomatitis [5, 16]. Necrotising gingivitis is characterised by marginal gingival necrosis, bleeding and pain (Fig. 1); necrotising periodontitis is an extension of necrotising gingivitis into the periodontal attachment apparatus with progressive looseness or loss of affected teeth [24] (Fig. 2); and necrotising stomatitis is an extension of necrotising periodontitis beyond the mucogingival junction with the necrotising inflammatory process spreading into labial, buccal, lingual and palatal mucosa [25] (Fig. 3).

In the presence of risk factors such as severe malnutrition and immunosuppression, the necrotising process of necrotising stomatitis then may spread rapidly devouring local tissues causing necrotising fasciitis, myonecrosis and osteonecrosis. As noma is preceded by anaerobic bacteria-induced necrotising gingivitis/periodontitis/stomatitis, early diagnosis of and treatment of any of these noma precursors will prevent the development of noma. Treatment regimen is simple and includes appropriate antibiotics, analgesics, frequent use of antibacterial mouthwash and gentle brushing to remove the dento-gingival plaque [5, 18, 22]. Importantly, any underlying risk factors such as immune suppression owing to HIV, malnutrition, malaria, typhoid, tuberculosis and viral infections must be attended to without delay [26].

### Acute noma

**Fig. 4:**
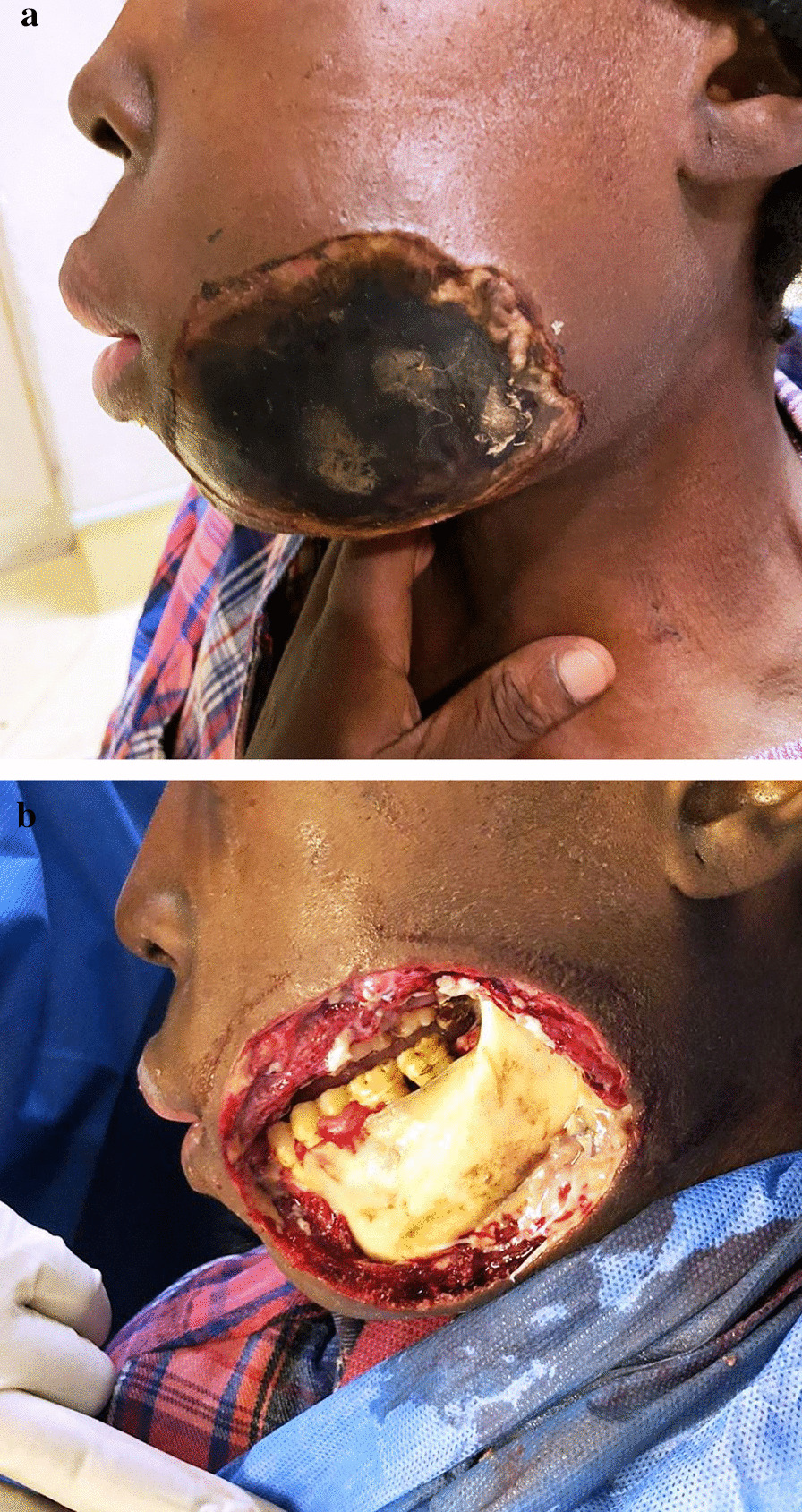
** a**, **b** Acute noma in malnourished impoverished juvenile: (**b**) and the arrested noma shortly after removal of the full-thickness necrotic buccal/facial tissue. Surgical reconstruction poses a major challenge.

The term ‘acute noma’ should be applied only when necrotising stomatitis and fasciitis are aggressively progressing to myonecrosis and osteonecrosis, recognisable by gross oedema, maceration and friability of the affected tissues, and with bluish-grey discolouration of the overlying skin. The affected tissues will then rapidly slough, with dehiscence of the necrotic bone, leaving the full-thickness circular or irregular defect characteristic of noma (Fig. 4a, b). When it occurs, the progression from necrotising stomatitis to the full-thickness destruction of noma is very rapid, often taking just a few days, and without urgent treatment most affected subjects die from septicaemia, dehydration and/or malnutrition [5, 16].

**Fig. 5:**
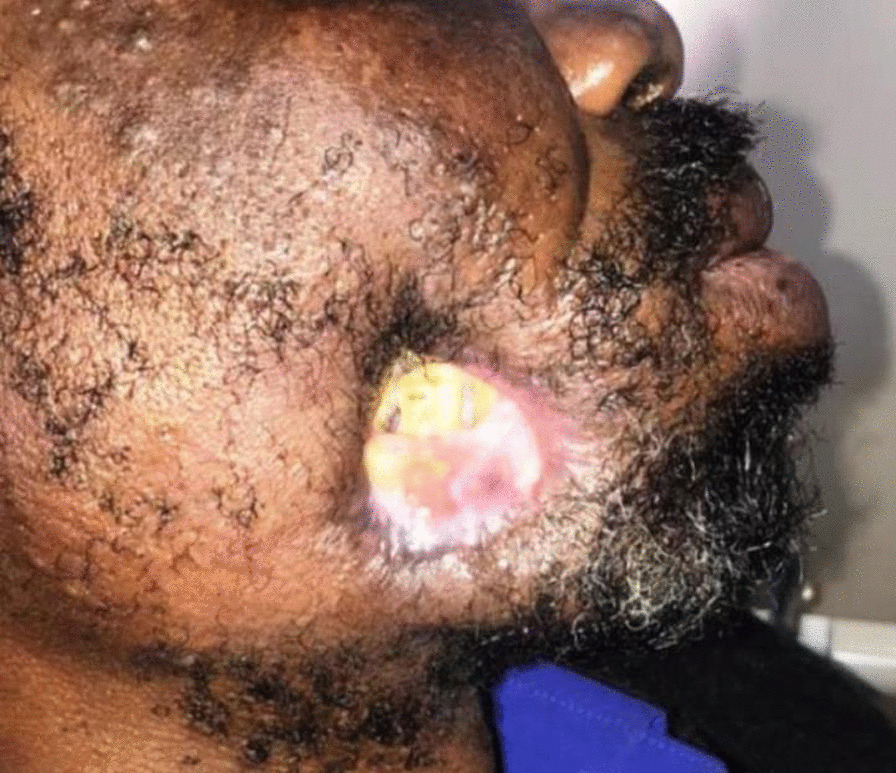
Arrested noma in a mature HIV-seropositive male, about 9 months after acute noma of cheek, before reconstructive surgery. The whitish material is food and tissue debris

Once necrotising stomatitis has progressed to acute noma, a hospital treatment protocol similar to that outlined above for necrotising stomatitis should be urgently and vigorously introduced, and the general condition of the patient be closely monitored [16, 19, 20]. In survivors of noma, the margins of the perforating, often extremely extensive lesions heal with severe scarring, resulting in functional impairment, and invariably in dreadful disfigurement (Fig. 5), with its associated psychological trauma and adverse, devastating social consequences [19].

It is important to note that necrotising gingivitis, necrotising periodontitis and then necrotising stomatitis always precede noma; but each of these is a controllable disease entity in itself, that under certain environmental, nutritional, systemic health and local intra-oral circumstances have the potential sequentially to progress to noma; nevertheless, they but rarely culminate in, and therefore should not generally be regarded as stages of noma.

While the occurrence of necrotising gingivitis, on a background of immune impairment and persistent physical or mental stress is relatively common, the incidence and prevalence of necrotising periodontitis is not common, of necrotising stomatitis is rare, and of noma is very rare [5, 16]. These necrotising oral diseases should not be termed ‘stages of noma’ but rather inflammatory phases which have the potential to culminate in noma, but this is a very rare clinicopathological event.

It would be wrong therefore, when trying to determine the prevalence and incidence of noma, to include necrotising gingivitis, necrotising periodontitis and necrotising stomatitis as stages of noma, and thus to qualify them to be regarded as noma in relevant epidemiological studies. This would make a mockery of the data about noma.

In our view, the usual term ‘acute necrotising ulcerative gingivitis’ is inappropriate, firstly because there is no form of necrotising gingivitis other than ‘acute’; and secondly, the ‘ulceration’ is not a primary feature, but rather is secondary to, and descriptive of the bacteria-induced necrosis of the gingival margins [16, 27]. Therefore, the simpler term necrotising gingivitis is to be preferred [16, 28].

Similarly, the term ‘gangrene’, customarily used to refer to the damaged tissue in noma is also wrong, because the apparent gangrene is not primarily ischaemic, but is caused by immuno-inflammatory damage to the local gingival vasculature, with secondary ischaemia followed by necrosis. Also, the pattern of progression of the necrosis does not follow the pattern of the blood supply to the affected tissue, as is a cardinal characteristic of gangrene [5, 16]. Thus, noma is in fact an infective-necrotising and not a gangrenous disease.

Furthermore, to list ‘oedema’ as a ‘stage’ of noma [1, 21–23] is nonsensical because oedema is a component of any inflammatory response. One might as well, and equally incorrectly, list as stages of noma any of the other signs of inflammation: erythema, heat, pain and restriction of function. This ‘stage’ of noma, that the WHO-related booklets/publications and some noma-related articles refer to as ‘oedema’, could equally well be applied to necrotising gingivitis or necrotising periodontitis or necrotising stomatitis where there is local tissue swelling associated with the intense inflammatory/necrotic features described above.

To even further confound the matter, there are some noma-related publications that include in the stage-classification a ‘Stage 0: simple gingivitis’ [1, 23]. Common gingivitis can be described as mild inflammation of the marginal and papillary gingiva, in response to accumulation of dento-gingival bacterial plaque owing to inadequate tooth cleaning; and is characterised by gingival bleeding upon gentle mechanical probing or other mild insults to the gingiva, including chewing hard foods, unaccustomed tooth-brushing, and professional periodontal examination. The marginal gingiva may appear erythematous and oedematous, but there is no periodontal attachment loss; and gingivitis is reversible when the burden of dento-gingival plaque is reduced by correct regular tooth cleaning. The prevalence of bacterial-induced gingivitis amongst children and adults may substantially exceed 50%, often depending upon the socioeconomic circumstances of the people [29–31]. Therefore, to include simple gingivitis as a ‘warning signal’ or an initial stage of noma, when about every second person around the globe is affected by gingivitis, does not have scientific merit, and as a matter of fact is misleading and confusing.

### Noma: acute and arrested phases

As long as the above-mentioned WHO-published stages of noma are applied in the diagnosis of noma [7, 23], and in the accumulation of data about the natural clinical course, epidemiological features and laboratory findings of the disease, any information collected by researchers and healthcare workers will be grossly biased and thereby misleading. This will seriously hamper efforts to understand the disease and to formulate strategies and public health measures to prevent, treat, and reduce the incidence, and prevalence of, and suffering from noma.

Therefore, since noma is a single and essentially invariable disease entity, that does not share its features or characteristics with any other disease entity, it does not admit of any classification, nor indeed of staging that introduces irrelevancies (e.g. WHO 2020, above), so we propose a simple, logical and self-evident categorisation of noma:

- Acute noma comprising active necrotising-induced local tissue destruction including necrotising fasciitis, myonecrosis, osteonecrosis, blue-black colour of overlying skin (Fig. 4a), skin perforation and ultimately full-thickness circular or irregular shaped defect (Fig. 4b); and systemic signs and symptoms including dehydration, malnutrition, anorexia, diarrhoea, anaemia, septicaemia, fever and pain. The course of acute noma as described here is very rapid, taking just a few days to develop from necrotising stomatitis [5, 14, 16, 20, 23], and is followed by either death, or arrested noma.
- Arrested noma comprising a tissue healing phase and the sequela of acute noma (Fig. 5) which include some of or all the following: scarring, fibrosis, oro-nasal or oro-antral openings, loss of teeth, ankyloses of the temporomandibular joint, functional impairments, facial disfigurement, and psychosocial suffering, requiring complex surgical reconstruction or prosthetic replacement of facial structures, psychosocial support, and physio-occupational therapy. The complex surgical reconstruction and functional rehabilitation, the outcome of which is at best a compromise, are usually performed 6–18 months after the conclusion of acute noma when the local healing and fibrosis have taken place and the general health of the patient has been stabilised [16, 19, 20, 26].

As arrested noma constitutes a life-changing and lifelong burden, it is difficult to estimate the time for recovery of physical and mental well-being [5, 14, 16, 23].

## Conclusion

Necrotising gingivitis and necrotising periodontitis should certainly not be regarded as stages of noma, but rather as inevitable precursors. Necrotising stomatitis, however, can be viewed as a possible pre-noma condition, as it not infrequently displays some encroachment of the intra-oral mucosal necrotising process upon the deeper tissues.

Application of this realistic two-part categorisation (bulleted above) may lead to future gathering of more accurate, less misleading information about the epidemiological, clinical, and immunopathological features of noma; and may bring to light on as yet unknown factors in the pathogenesis of noma. It may also help to and explain why, implausibly, most cases of noma have been reported from certain sub-Saharan African countries, but far fewer from similarly underprivileged, disadvantaged communities in other countries. There is also a lack of knowledge or understanding of why only a very few malnourished or immunosuppressed persons, all living under the same conditions, and despite having established necrotising gingivitis/periodontitis, will develop noma. New data may well suggest new strategies to fight noma and to alleviate the suffering of noma survivors [19, 20, 32].

## Data Availability

Not applicable.
